# Anæsthetics in Dental Practice

**Published:** 1888-09

**Authors:** James E. Harris


### THE DENTAL REGISTER.

VOL. XLII.] SEPTEMBER, 1888. [No. 9.

### COMMUNICATIONS.

### Anæsthetics in Dental Practice.

BY JAMES E. HARRIS, M.D., D.D.S.

The history of anæsthetics in general, and of the different agents individually, is too well known and far too broad a subject for such a paper as mine must be; therefore, it is my intention to write only of the general employment of anæsthetics by the dentist.

The idea at present prevails that it is more dangerous to administer anæsthetics for dental operations than for general surgery, and perhaps, to a certain extent, there may be cause for it, but why? One strong reason is the great difference in the sequel to a death under the hands of a surgeon, and a death under the hands of a dentist. In the case of the surgeon, if he be a competent and conscientious man, he will be filled with much regret by the thought that such an accident has befallen him, but will console himself with the idea of duty properly fulfilled; and at the next meeting of his medical society will honestly report the case; and the following discussion will probably result in the promulgation of ideas which will prove of benefit to the profession at large and the public in general. A brief notice may appear in the local newspaper; the bare facts may be copied by a very few other papers, but this will be all, and soon the public has forgotten it. But with the poor, unfortunate dentist, how is it? Alas! far different, as a rule. The patient dies and the coroner comes; then follows the report of the case, which will probably occupy a goodly portion of a newspaper column, and the details will be copied and added to by other papers until it occupies nearly two columns and has the family history of dentist and patient also added and perhaps a picture of both. Our dentist is fortunate then indeed if he ever hears the end of it.

This comparison may be a little exaggerated, but only a little, and it is one of the ways in which the idea of anæsthetics being more dangerous in dentistry becomes popular. But there are other ways and how are we to overcome them? In the first place let our medical men be more charitable toward us, and let us, as dentists, exercise more charity towards each other, for we are too jealous and too ready to rejoice when a fellow-practitioner gets into trouble.

But the one great way is to prove it true that the danger is not greater and do it by administering only a pure article and in the proper way—which naturally means that the administrator must thoroughly understand his business; and right here is where our education is frequently at fault. This part of our training at college is too frequently neglected and we are turned adrift upon the world without ever having operated upon an anæsthetized patient and often without ever having seen an anæsthetic administered, unless possibly nitrous oxide gas. Such a man can not possibly be expected to have that first essential to a successful administrator, namely confidence; and if the giving of an anæsthetic should be, for any reason forced upon him, he tremblingly makes doubtful preparations, tremblingly folds his cone, and tremblingly applies it to his patient's nostrils, trying to think if he has not forgotten some precaution or done something amiss—then the exciting stage comes on and feeling sure he has made a mistake or done something wrong, he flings away his towel, afraid to proceed further and we have a patient and dentist both thoroughly demoralized—either this or he is afraid to push his anæsthetic to its proper extent and operates too soon, which is equally demoralizing in its results, to say nothing of the danger consequent upon such a course.

What then is the proper method of administering an anæsthetic? Certain precautions must be followed and I do not think I can do better than to quote five simple rules as given in a paper by Dr. J. J. Chisolm, Professor of Diseases of the Eye and Ear in the University of Maryland.

1st. "I always, without a single exception, give a strong drink of whisky, from one to two ounces, to every adult to whom I intend to administer chloroform. This is done a few minutes before they get on the operating table. Because I never omit this fundamental law, and in advance sustain the heart's action against the depressing effect of the anæsthetic, in not one of my twelve thousand cases have I ever had to use, in a single instance, a hypodermic of whisky. It is already in the stomach, should it be needed and can do no harm if not required.

2nd. Always loose the neck and chest clothing, so as to have no impediment to respiration.

3rd. Only administer chloroform in the recumbent posture, with the body perfectly horizontal and head on a low pillow, this pillow to be removed as anæsthesia progresses.

4th. Give chloroform on a thin towel folded in conical form, with open apex, so that the vapor before inhalation, will be freely diluted with atmospheric air. In holding the cone over the face of the patient, at some little distance from the nose, place the fingers under the borders of the cone, for the double purpose of allowing air to enter freely and also to prevent the chloroform liquid on the towel from coming in contact with the skin of the patient's face, and thereby avoid its blistering effects.

5th. Should loud snoring occur, force up the chin. This manipulation, by straightening the air-passage from the nose to the larynx, makes easy breathing. The forcible elevation of the chin is far better in every respect than pulling out the tongue. It is easier of application, more quickly done, requires no instrument, and is much more efficient in removing the impediment to respiration."

In the same paper Dr. Chisolm says: "The recumbent posture I consider essential for the safe administration of any anæsthetic, whether it be chloroform, ether, or ethyle; hence these agents are not safe remedies at the hands of dentists, who place their patients in a sitting posture."

But this is exactly what we should not do. Admitting that his rules are simple and most excellent ones to follow, let us briefly see if there is any reason why we, as dentists, cannot fully comply with them.

1st. Give whisky—easy enough.

2nd. Loose clothing--equally as easy.

3rd. Only administer chloroform (or any anæsthetic) in the recumbent posture--a most important rule and one that should never be violated by any one, for when necessary a competent dentist should be able to extract almost any tooth in the mouth while the patient is lying at full length on the back.

4th. Give chloroform on a thin towel, folded in conical shape, with open apex--this is usually done by every one, or should be.

5th. Should loud snoring occur, force up the chin--if the right kind of chair be used this can be done with ease. But what is the right kind of chair? I do not remember ever having seen but one of them and it is now in the extracting room of the Dental Department of the University of Maryland. It is a very old pattern and I do not know by whom it was originally manufactured. It has its back hinged to the seat and held in place by ratchets caught in the arms. By lifting the rachet the back can be lowered until it is almost on a level with the seat, thus putting the patient in the position designated by rule 3. The head-rest is hinged to the back and also regulated by a ratchet, so that it can be elevated sufficiently to take the place of the "low pillow" and "as anæsthesia progresses," can be lowered sufficiently to compensate for removal of said pillow, and "if loud snoring occurs" the rest can be allowed to drop away from the head entirely, letting it hang over the edge of the chair with the chin forced upward thus straigtening "the air-passage from the nose to the larynx." Another advantage of this chair is that while the patient is thoroughly anæsthetized, he can if necessary, be put in any desired position, even an upright one, by simply raising the back, and the rachet will catch and retain it at any elevation to which it is placed. It is often quite convenient to elevate the patient a little, but the chair should be made perfectly flat again as soon as the operation is completed.

Suppose we are about to give a patient an anæsthentic and extract several teeth. The patient is seated in the chair with the clothes loosened; if chloroform or ether is to be employed and the patient an adult, give a full dose of whisky; if nitrous oxide gas or bromide of ethyl is to be used, or the patient is a child no whisky is needed. Next lower the back of the chair, putting the patient in the recumbent position and we are ready for the anæsthetic but what shall it be? Nitrous oxide is probably the safest but some of its characteristics are objectionable features. First its anæsthetic effect is often so transient that only a few teeth can be extracted, and where many are to be removed the patient recovers from its influence before the operation is complete, and, as I've often heard them do, use strong language in voting the gas a fraud and the patient vows he suffered as much as though he had taken nothing at all.

Sometimes, the patient after many misgivings, has screwed his courage to the sticking-point, and when only partially anæsthetized will get perfectly wild, grab the inhaler from his mouth, and jump up in a fearful state of nervous excitement, quite determined to take no more. Had it been possible to have forced the agent on him and completed the operation he would, in all probability, thankfully have acknowledged it, but I defy any one to successfully administer gas against the patient's will. A third objection is that we occasionally meet with some one whom it is almost impossible to anæsthetize with gas; so it is not always to be depended upon and especially if the operation will occupy any considerable time we will find ether or chloroform better.

The best manner of giving ether is from a funnel-shaped sponge; as before stated, chloroform should be given upon a towel folded into a cone with apex open. As regards safety, I have been led to believe that there is no great choice between these agents, provided each is properly administered. Ether may be given more rapidly and to the exclusion of atmospheric air; but with chloroform the safety of the patient greatly depends upon a proper admixture of air with the chloroform vapor. In the beginning chloroform should be administered slowly, but as the exciting stage comes on it may be given a little more rapidly.

Ordinarily the operator should not administer the anæsthetic himself, but trust it to another who should stand behind the patient, holding the inhaler in one hand and having the fingers of the other upon the temporal artery. The operator stands on the right side of the chair, and an assistant on the left. Should the patient become violent these two can manage the hands and body, and the administrator can let an arm rest on either side of the head, thus keeping that under control.

If the operation to be performed is short and simple, the bromide of ethyl is undoubtedly one of our best agents, and when properly used it is certain, rapid, not nauseating, and the patient recovers promptly and thoroughly, without any of that stupor which so often follows chloroform or ether. But to use it properly certain directions must be remembered and carried out, and here I again quote Dr. Chisholm, who has so successfully used this agent in eye surgery:

"The best inhaler for the giving of the bromide of ethyl is a thick towel folded into the form of a small cone with closed apex. Between one of the folds of the towel I place a sheet of paper which makes the cone nearly air tight. The base of the cone must be wide enough to enclose both mouth and nose. The soft material of which the inhaler is made enables the rim to be kept firmly in contact with the face so as to exclude air from entering. I always instruct the patient how to make long inspirations, and inform him that he must do this notwithstanding the fact that he will feel somewhat stifled. I also try to give him confidence by assuring him that a very few inspirations will put him to sleep. Usually I make him go through the process of strong respiratory movements in advance so that he will know exactly how to proceed. Into this towel cone I pour about one drachm of the bromide of ethyl and immediately invert the inhaler over the nose and mouth of the patient, holding its edge down firmly over the face. There is no fear of creating asphyxia, as all air can not be excluded, and the height of the cone makes a considerable air-chamber into which the patient breathes.

Children usually struggle to escape from the apparatus. *The cone, however, must not be removed from the face for an instant until anæsthesia is produced.* At first some patients will resist the breathing of the vapor, but there is no fear that they will not catch their breath in time. Should children cry it only increases respiratory efforts, which the more surely and quickly will bring about the introduction of the vapor into the lungs. As a rule a dozen full respirations are all that are needed to produce deep narcosis.

I recognize this desirable condition by a stoppage of all struggles. I have had deep sleep brought on by the sixth inspiration, when complete relaxation ensues with quiet breathing and an absence of reflex irritation should the conjunctiva be touched. The patient retains the usual heathy color of lips and cheeks as if in ordinary sleep, and the pulse becomes slower and stronger as the narcosis becomes profound. Thirty seconds as a rule, is sufficient to bring about this desirable condition and have the patient ready for operation. I have not found this anasethetic sleep last more than two or three minutes, often not so long. Usually the patients awake suddenly and as completely as they would from ordinary sleep. They are able to get down from the operating table without assistance and walk off without staggering, and with brain clear to answer correctly any question, "in fact, quite themselves."

If chloroform or ether is given on a full stomach vomiting will almost certainly result; therefore, when possible select a time about three hours after a meal; then the stomach will be empty and yet the patient will not be weakened by fasting. During administration we should be always watchful for any signs of emesis and at the first indication of it, turn the patient's head on one side, so that the vomited matter may escape from the mouth; otherwise some particles may get behind the epiglottis and produce suffocation.

When the patient is sufficiently anaesthetized, as shown by insensibility to conjunctival irritation, the administrator resigns his position at the rear of the chair to the operator, who, if the teeth to be extracted are in the lower jaw, lifts the back of the chair slightly and pushes up the head-rest to its fullest extent, thus forcing the patient's chin against the breast. By this plan the light is allowed to fall directly into the mouth and the position is the one most favorable for the extraction of these teeth.

One of the reasons why anæsthetics are more dangerous in dentistry is from blood getting into the air-passages, but with the patient's head in the position I have just described, the floor of the mouth forms a cup which holds the blood and when the operation is completed the head should be turned on the left cheek with the face slightly downward and hanging over the side of the chair when the blood will run out of the mouth. To extract teeth in the upper jaw, the back of the chair often need not be moved and the head-rest not at all; but lift the head and place it with chin slightly up, then begin with the most posterior tooth on the left and come forward as far as the cuspid; then turn the head on the left cheek, face slightly down, and in this position every remaining tooth in the upper jaw can easily be extracted, while the blood is running out of the mouth and not down the throat.

This theory is good and practicable, for I have often seen it followed out and have adopted it myself. In extracting it is always best to extract the lower teeth before the upper and the posterior before the anterior, for the blood will then flow out of the way instead of in it. It may be well to have the assistant on the left provide himself with a basin of warm water and several small soft sponges, with which he can wipe out the patient's mouth as necessary.

Now let the patient lie quietly and he will soon return to consciousness, or if he seems slow to do so, fresh air or a little water sprinkled in the face, or a few inhalations of ammonia will revive him.

We will sometimes be annoyed and inconvenienced by the weakness and stupor following revival from ether and chloroform and it is for this reason that I give such decided preference to the bromide of ethyl—from which the patient recovers promptly without nausea and within a few minutes after the ethylization is able to leave the office with perfect comfort and a clear brain.

But regardless of the anæsthetic chosen for our purpose, I think I have made it sufficiently clear that by the use of a suitable chair, a rapid and skillful style of operating, and by employing only the proper position, anæsthetics are quite as safe in dental practice as in general surgery. I am thoroughly convinced that this is true and do not believe it can be disproved.

What are the contra-indications to the use of an anæsthetic? In my opinion almost none, unless it happens to be the absence of any one who knows how to properly administer it. I was once taught, like many others, that any disease of heart, brain, lungs, etc., contra-indicated the administration of an anæsthetic but this I never fully believed, for I felt sure that if affections of the brain were fatal many administrators would die from the fumes inhaled while giving it to others. I have seen it given to too many patients with the diseases supposed to contra-indicate its use.

I surely may be pardoned if I again quote Dr. Chisholm, whose surgical experience of over thirty years has been the subject of public professional observations.

He says: "My rule of practice has always been to do surgical work with the least possible pain, and to refuse anæsthetics to no surgical patient. Those patients were of all ages from infants to octogenarians, and represented every condition of disease and health. Some were strong, and some were feeble, with lung, heart, and kidney diseases. No pathological lesion in any other part of the body deters me from the use of chloroform should an eye operation be required. Cataract patients are usually old; most frequently exhibit decided senile changes. I suppose an average of sixty years of age would represent this class of patients; seventy, seventy-five, to even eighty-five, ninety, and ninety-five, are at times the respective ages; ninety-six is the extreme age at which a successful cataract extraction has been performed by me under chloroform. It is well known that fatty hearts are very frequently found in old subjects in dissecting rooms. My cataract operations now reach fifteen hundred. Of these many must have had fatty hearts."

In the same article Dr. Chisholm says: "Had death from chloroform occurred in my practice it would have received, necessarily, prompt publicity. That I have never had one, and that I have never refused chloroform to any patient received into the hospital for surgical treatment is a fact well known." Surely such positive evidence from such a man is worthy of the utmost consideration from all who use anæsthetics. But in spite of our best precautions dangerous symptoms will sometimes supervene, and how are we to meet them?

During anæsthesia death may result from failure of heart's action, respiration continuing; failure of respiration, heart's action continuing; or we may have both at once. In any case it may be well to administer nitrite of amyl. This agent increases the action of the heart by paralyzing its inhibitory nerve supply, and by its influence upon the vaso-motor system, increases the calibre of the arteries of the brain, thereby promoting the facility of the return of blood to this organ, which is what we most desire. But the means to be most surely relied upon is the inversion of the patient, and do this at once. Do anything else you like, but first invert the patient. The experiments of Nelaton, that well-known French surgeon, are too familiar to need repetition. They prove that in chloroform narcosis, the respiratory and cardiac centres are weakened by the anæmic condition of the nervous apparatus. So, it must be the horizontal position which is fatal, and if we want to save valuable lives when threatened by anæsthesia, by all means let us hang up our patients head down, and the return of blood to the weakened centres will be sufficient stimulation to start heart and lungs going again.

Dr. Chisholm reports four cases in which he feels sure the patient's lives were saved by inverted suspension, and by that alone.

The late Dr. J. Marion Sims reported before the British Medical Society, in 1874, the case of a young and titled Frenchwoman, upon whom he operated in the presence of the distinguished Nelaton and others. Chloroform was used and at three different times the patient was apparently dead. But each time, under Nelaton's direction, she was suspended and her life saved.

Of course there must be many such cases, but the last I know of was reported by Dr. Jay, of Indiana, before the Mississippi Valley Dental Association, in March, 1888. In his case ether was given by patient's physician, and the teeth were successfully extracted, but instead of recovering the patient began to breathe less and less, and finally stopped altogether. Suspension was resorted to with various other means, and she recovered and relapsed five times, but after the sixth suspension and recovery, went on to her normal condition. The case is fully reported in May number of the *Ohio Journal of Dental Science*, and is worthy of perusal, but I am confident that if suspension alone had been used the patient would have recovered equally as well.

The other means for resuscitation at our command—fanning, fresh air, water-dashing, whisky or ether injections, electricity, artificial respiration, may all be well enough in their way, but I doubt their efficiency if the patient be left in the horizontal position and general experience has, unfortunately, too often shown this to be true. When we have suspended animation all of these means may be tried, but may also be useless if we will at once hang up the patient by the feet without loss of time and allow the blood to flow back to the anæmic head and heart and re-stimulate the nerve-centres before the vital spark is altogether extinguished. But vigilance and promptness must be insisted upon, for a fire cannot be rekindled by adding fuel if there be no live coals in the grate.

In conclusion then, let us bear always in mind that our anæsthetics are powerful agents for evil as well as good, and that we can kill almost any patient by abusive or careless administration. Let us also remember that too little of the anæsthetic, or not enough to protect the important vital centers from the evil influence of painful reflex action is equally as dangerous to our patients as an overdose of the narcotic inhalant, and what we most require for successful administration is skill, care, prudence, judgment, and confidence, and sufficient courage and presence of mind to do the right and proper thing for the welfare and safety of our patients in time of need.

If we, as dental surgeons, will remember and do all this, then beyond the shadow of a doubt, our record must be as clean and as free from reproach as that of any other surgeon.

